# Toward developing adolescent-centered machine learning methods to detect depression: Interviews with Latino adolescents to identify signals of emotional and somatic symptoms within social media data

**DOI:** 10.1371/journal.pdig.0001178

**Published:** 2026-01-02

**Authors:** Celeste Campos-Castillo, Prathyusha Galinkala, Katherine Craig, Linnea I. Laestadius

**Affiliations:** 1 Department of Media and Information, Michigan State University, East Lansing, Michigan, United States of America; 2 Department of Communication, Michigan State University, East Lansing, Michigan, United States of America; 3 Department of Sociology, University of Colorado Boulder, Boulder, Colorado, United States of America; 4 Zilber College of Public Health, University of Wisconsin-Milwaukee, Milwaukee, Wisconsin, United States of America; North Carolina A&T State University: North Carolina Agricultural and Technical State University, UNITED STATES OF AMERICA

## Abstract

Despite rising use of machine learning (ML) methods to detect depression within social media data, few are developed with and for adolescents. This is unfortunate, because adolescents may be more likely than adults to experience somatic than emotional symptoms and may be less likely to express emotions on social media. Accordingly, ML methods that focus on emotional symptoms may undercount adolescents at risk for depression. As a step toward developing an adolescent-centered ML method, we co-developed an interview guide with Latino adolescents to understand 1) social media norms for expressing somatic and emotional symptoms; and 2) identify potential signals of each. For the latter, we adopted a novel approach of asking interviewees to take on the “human classifier” role and tell us what they look for within social media data. Using framework analysis on 43 interviews with Latino adolescents, we find evidence suggesting norms prescribe more strongly against conveying emotional symptoms than somatic symptoms on social media. Additionally, rather than literal statements conveying they are experiencing depression, adolescents appear to use audiovisual cues to signal emotional symptoms and posting behavior (time of post, posting less) for somatic symptoms. Accordingly, norms may hinder opportunities for leveraging social media data to detect depression among adolescents, particularly when using ML methods that search for literal statements of depression or signals of emotional symptoms. Because peers tend to recognize depression in an adolescent earlier than medical experts, these findings suggest the need to develop and validate ML methods that incorporate a set of signals for somatic symptoms, particularly audiovisual cues and posting behavior. We discuss the benefits of “centering at the margins,” which is focusing on a population that is understudied within this domain, and the need for ML methods developed with adolescent input.

## Author summary

Adolescents tend to experience depression differently from adults and appear to express it differently on social media as well, suggesting the need to work toward developing machine learning (ML) methods to detect depression that are attuned to them. As a step toward this, we took an adolescent-centered approach by interviewing Latino adolescents to understand 1) social media norms for expressing somatic and emotional symptoms; and 2) identify potential signals of each by asking them what they look for when determining whether a peer may be experiencing each set of symptoms. We find evidence suggesting norms prescribe more strongly against conveying emotional symptoms than somatic symptoms on social media. Additionally, rather than literal statements conveying they are experiencing depression, adolescents appear to use audiovisual cues to signal emotional symptoms and posting behavior (time of post, posting less) for somatic symptoms. We suggest developing ML methods that focus on adolescents by incorporating a set of signals for somatic symptoms, particularly audiovisual cues and posting behavior.

## 1 Introduction

Worldwide, rates of depression among adolescents (12–19 year-olds) have risen in recent years [1]. In the U.S., 1 in 5 adolescents have experienced symptoms of depression [2]. Adolescents from minoritized ethnoracial groups face the additional burden of overcoming barriers to accessing mental health treatment [3]. Compared to their White counterparts, they are more likely to experience clinician bias, have weaker adult support, and have access to fewer economic resources to prioritize treatment over other household needs [4–6]. Where adolescents from minoritized groups outpace their White counterparts is in emergency department visits for mental health concerns [7], suggesting their mental health concerns are more commonly unaddressed until a crisis arises.

Innovations are needed to improve identifying signs of depression within adolescents, and ideally in early stages. Alongside rising rates of depression has been an increased interest in using artificial intelligence, including machine learning (ML) methods to automate detecting depression within user-generated data, such as social media posts [8–13]. ML methods detect patterns within data, referred to as signals, to categorize the users who generated the data into groups with meaningful labels, such as “depressed” and “not depressed.”

Despite the enthusiasm for ML methods, there remain limitations that curb their potential for mitigating rising rates of depression among adolescents. Most ML methods use data from young and middle-aged adults or target platforms (e.g., Twitter) that are frequented less by adolescents than adults [8–13]. The disparity in attention is important because adolescents experience depression differently from adults [14,15] and therefore likely express it differently on social media [16]. Adolescents also have distinct norms about what they share about themselves online [17,18], which may further distinguishing their online expressions from those of adults. The process of categorizing based on signals that may not be tuned for adolescents can result in algorithm bias, limiting the utility of models and presenting potential health risks from implementation.

As a step toward addressing limitations in extant ML methods, we present an adolescent-centered approach in which we partnered with adolescents to identify potential signals on social media that indicate depressive *symptoms*. Because people experience symptoms, not diagnoses [19], and because the labels used to refer to symptoms can vary cross-culturally [20], the approach seeks to identify signals of *symptoms consistent with depression*. Thus, the labels used to categorize are no longer “depressed” versus “not depressed,” but rather “symptomatic of depression” versus “not symptomatic of depression.” We build on related work that also focuses on symptoms rather than diagnostic-like labels for developing ML methods [21–23] by acknowledging that what constitutes “symptomatic” varies across a population, such as by age group. Specifically, prior work suggests that depression comprises emotional (e.g., sadness) and somatic (e.g., fatigue) symptoms, with the latter more common among adolescents than adults [14,15]. Thus, developing an approach focused on identifying signals of symptoms consistent with depression that also parses out type of symptoms would be a critical step toward improving detection of depression among adolescents. Such an approach may, for example, give greater weight to signals of somatic symptoms over those for emotional symptoms. Moreover, a focus on symptoms rather than a diagnostic-like label may enhance earlier detection and access to mental health support, because experiencing symptoms consistent with depression that do not cross the threshold for a diagnosis can still be consequential for individuals [24].

In our adolescent-centered approach, we “centered at the margins,” meaning worked directly with those who are impacted by yet are traditionally excluded from power structures [25], specifically Latino adolescents. Our focus on Latino adolescents is both to address social justice concerns about their lack of representation in research on technology and mental health and an analytic strategy: while studies show somatization is more typical among adolescents than adults [14,15], it is particularly pronounced among Latinos [26,27]. We co-developed an interview guide with Latino adolescents, which we used to ask other Latino adolescents what *they* look for on social media to determine who may be experiencing symptoms consistent with depression. In other words, we asked participants to take on the role of a human classifier to generate a list of potential signals of emotional and somatic symptoms that are consistent with depression.

The interview guide was developed to answer two interrelated research questions: RQ1) What norms shape whether Latino adolescents convey emotional and somatic symptoms consistent with depression on social media? and RQ2) What signals do Latino adolescents use to identify emotional and somatic symptoms of depression among peers on social media? Given prior work suggesting a reluctance among adolescents to post about depression on social media [28] and privacy concerns that limit with whom they may share such content [5], RQ1 is important for determining the likelihood that data scientists would be able to find and access potential signals of emotional and somatic symptoms within social media data. RQ2 identifies the signals within social media data that may be useful for detecting the two sets of symptoms. Through contextualizing signals within the norms discovered from RQ1, it may reveal further information about which signals may be most likely to appear within social media data and thereby be most useful for developing ML methods tuned to adolescents. After reviewing relevant work, we describe further the development of the interview guide and summarize findings from 43 Latino adolescents who completed the interviews. Based on findings, we make recommendations for working toward developing and validating ML methods to detect symptoms of depression among adolescents.

## 2 Related work

### 2.1 ML methods to detect depression within social media data

Several ML methods, and more advanced deep learning methods, have been developed to create mental health profiles of individuals by using their social media data [8–12]. Signals typically comprise the text of posts made by users but can also include emoticons, images, and sound in the post, while other signals used include descriptors of the users, such as their gender and age. Examples of “ground truths” used to verify how accurate ML methods can detect who may be depressed within social media data are medical diagnoses and self-reports of users, such as users’ participation in social media groups for those with depression, their declarative statements in posts that they are experiencing or were diagnosed with depression, and their responses to screeners for depression (e.g., the Patient Health Questionnaire, Center for Epidemiological Studies Depression scale). Other examples rely on annotation, typically involving research team members assigning whether a post is or is not indicative of depression. While crucial steps toward detecting depression, the stigma of depression can introduce biases, because stigma creates barriers to accessing medical care for depression, receiving a medical diagnosis, and declaring to others that one may be experiencing depression [29].

As a step toward addressing these limitations, a subset of ML methods have been developed to detect symptoms consistent with depression within social media data. The profile of indicative symptoms may come from screeners for depression (e.g., the Patient Health Questionnaire, Center for Epidemiological Studies Depression scale) or from identifying a set of features in a post representing mental states that tend to co-occur among those users labeled as depressed [21]. While promising, these tend to focus on emotional rather than somatic symptoms, which limits transferring the methods to adolescents, who are more likely than adults to experience somatic symptoms [14,15]. Notable exceptions are studies by De Choudhury and colleagues [22], which included posting behavior (posting during twilight hours, posting less often) as signals reflecting somatic symptoms, and Mowery and colleagues [23], which annotated Twitter posts based on the presence or absence of symptoms that included somatic symptoms. However, both studies show the presence of emotional symptoms was more common than of somatic symptoms in posts categorized as indicating depression, which may reflect the sample being comprised primarily of adults [22] or the posts being annotated by adults [23].

We build on this prior work focusing on symptoms by taking an important step toward modifying the approach for adolescents by asking them about the signals within social media that indicate symptoms consistent with depression. Asking adolescents directly is an important step given research suggesting both the experience and expression of depression differs between adolescents and adults. Future work may then validate the suggested signals and compare them to others typically used, such as a medical diagnosis, to refine existing ML models.

### 2.2 Depression within adolescents

Scholars recognize that the experience of depression is dimensional, with two typically appearing across a range of screeners and diagnostic interviews [30]: emotional and somatic symptoms. Studies show somatic symptoms are more typical among adolescents than adults [14,15], particularly among Latinos [26,27]. Somatic complaints appear critical for early detection of depression among adolescents, in that high levels of somatization among those initially categorized as nondepressed is predictive of their subsequent categorization as depressed via screeners and diagnostic interviews [14,31,32]. Accordingly, ML methods that focus on explicit statements of “depression” or only on emotional symptoms risk being biased against adolescents in that they may undercount those needing mental health support.

A more basic question that has, to our knowledge, not been asked, is whether it would be practical to use social media data to detect depression among adolescents. This is an important question to ask, given related work suggesting adolescents may avoid posting about their depression to avoid being seen as “attention-seeking” or “sadfishing” [28,33]. Further, adolescents may prefer to not share emotional symptoms on social media or may do so to only a subset of their online networks (e.g., only to their close friends), indicating such signals would be rare or absent in their data. Prior work shows there is a tendency to prefer making posts on social media that connote positive (e.g., joy) over negative emotions (e.g., sadness) [33]. Accordingly, not only do adolescents experience depressive symptoms differently from adults, but there is reason to believe they may also express them differently from adults on social media, which raises the need to develop ML methods tuned with adolescents in mind.

Both age and gender of adolescents may further shape experiences and expressions of depression on social media. Somatization of depression is more likely among younger adolescents and girls [31,34,35], suggesting that including signals of somatic symptoms is critical for detecting depression within these groups. Regarding expression, the relevant literature does not suggest potential patterns. On one hand, masculine ideals dictate that emotional expression is more appropriate for boys than for girls [36,37]. On the other hand, some studies suggest boys are more likely than girls to share their thoughts and feelings on social media [38]. Understanding potential demographic patterns will be critical for developing accurate ML models.

Taken together, prior work indicates an emphasis on developing ML methods that categorize who may and may not be depressed, with some attempts to detect depression via detecting symptoms consistent with depression. The latter approach is ideal for developing ML methods for adolescents, because they tend to experience depressive symptoms differently from adults and can therefore go undiagnosed [14,31,32]. A focus on symptoms may also enable earlier detection of who may need support [24]. Moreover, it is important to consider norms prescribing the sharing of depressive symptoms among adolescents, because such norms may limit the utility of implementing ML methods tailored for adults on their social media data. Lastly, both age and gender may shape patterns in the experiences and expression of depression on social media, and these appear pronounced among Latino adolescents. These issues give rise to our research questions, which we addressed with the current study: 1) What norms shape whether Latino adolescents convey emotional and somatic symptoms of depression on social media? and 2) What signals do Latino adolescents use to identify emotional and somatic symptoms of depression among peers on social media?

## 3 Methods

### 3.1 Research setting

In this adolescent-centered design, we partnered with a non-profit organization in Milwaukee, WI that serves the Latino community (comprised primarily of those of Mexican origin) to recruit Latino adolescents between the ages of 13 and 17 who use social media at least once per week. The organization screened potential participants based on the eligibility criteria.

### 3.2 Research ethics

The Institutional Review Board at the University of Wisconsin-Milwaukee approved the research as an exempt study (#19.A.184). Adolescents and guardians provided written assent and consent (respectively) before data collection began. Both assent and consent documents were available in English and Spanish. Participants who enrolled in the study received a $40 Amazon Gift Card.

### 3.3 Community outreach

Before conducting interviews, we worked with the organization to convene a one-hour focus group of 15 Latino adolescents between the ages of 13 and 17 to obtain feedback on our interview questions. The focus group was led by the first author, a Latina faculty researcher, and attended by the third and fourth authors, both of whom are White women. We shared a draft of the interview guide and asked them to think out loud as they read and interpreted the meaning of the questions. To identify how best to describe symptoms of depression, we gave each adolescent an index card and asked them to write descriptors of depression. Each adolescent shared what they wrote and we asked the group to collectively reflect on the descriptor. At the end of the session, we collected the cards to incorporate them into the interview guide.

### 3.4 Interview questions

In consultation with a licensed clinical psychologist who works in pediatric settings, we used the cards collected from the focus group to design interview questions to ask about how adolescents express signs of emotional and somatic symptoms and when doing so was appropriate (if ever). The resulting questions are shown in Table 1.

**Table 1:** Interview questions.

Research Question	Emotional	Somatic
1. What norms shape whether Latino adolescents who may be experiencing depression convey emotional and somatic symptoms on social media?	When people feel bad, they sometimes feel anxious, isolated, really sad, or like they want to give up. If you were feeling this way, how would you decide whether or not to show this on social media? When do you think it is ok (and not ok) for your friends to use their accounts to show they are feeling this way?	Sometime people also feel physically drained, like they’re exhausted, have lost interest in things they usually care about, or have trouble going to sleep or staying asleep. If you were feeling this way, how would you decide whether or not to show this on social media? When do you think it is ok (and not ok) for your friends to use their accounts to show they are feeling physically drained?
2. What signals do Latino adolescents use to identify emotional and somatic symptoms of depression among peers on social media?	How can you tell from a friend’s social media accounts that they might be feeling anxious, isolated, really sad, or like they want to give up? Can you describe an example of a time you thought a friend was feeling this way?	How can you tell from a friend’s social media accounts that they might be feeling physically drained? Can you describe an example of a time you thought a friend was feeling physically drained?

The interview asked about emotional symptoms, followed by somatic symptoms, with phrasing designed to reflect the descriptors from the index card task. For each set of symptoms, we first asked questions to address RQ1, which is about understanding the norms for expressing the symptoms on social media. We provide a scenario, which is suitable for eliciting norms [39], and then asked interviewees about the appropriateness of self-posting and the appropriateness of a friend posting. Questions about the self and the friends access personal and social norms, respectively, and shape decisions to post [17,18]. We then asked questions to address RQ2, which is about identifying potential signals of emotional and somatic symptoms within social media data. The questions asked interviewees to take on the role of human classifier and describe what they look for in others to identify each symptom set. We also asked them to describe an example of when they classified (“thought”) a friend may be experiencing a set of symptoms.

### 3.5 Interview

The semi-structured, in-person interviews were held in a quiet room located in the community organization. The three researchers who conducted interviews were all female and trained in either sociology or clinical psychology. One was Latina, a second was Asian, and the third was White. Although a Spanish-speaking interviewer was available, all interviews were conducted in English at the request of the adolescents. Prior to starting the interview, the interviewer described the procedures and confirmed assent from the adolescent. Once assent was confirmed, the participants completed a demographic survey and selected a pseudonym. Interviews were audiorecorded and lasted approximately 60–90 minutes. Recordings were transcribed verbatim by a professional contractor. At the completion of an interview, the interviewer compensated the participant, provided them with information about local mental health resources, escorted the participant to the main lobby, and then wrote a memo summarizing the interview and environment.

### 3.6 Analysis

An interdisciplinary team of female researchers with expertise in human-computer interaction, public health, and sociology worked together to analyze the data using framework analysis [40]. The defining feature of framework analysis is the matrix that researchers develop to summarize the data across interviews. We structured our matrix by research question and by set of depressive symptoms. The first two authors began by open coding the first 4 interviews to identify initial codes and develop the codebook. Following the creation of the initial codebook, the second author coded the remaining interviews. The first two authors met weekly to discuss the codes, perform the constant comparison method to revise the contours of each code [41], and generate the matrix. The analysis was performed using MAXQDA 2024. The full team discussed the findings and implications.

## 4 Results

We conducted interviews with 43 adolescents. A majority identified as girls (n = 25) with the remaining identifying as boys (n = 18). Participants’ ages ranged from 13 - 17, with a modal age of 15. Pseudonyms chosen by participants are used to label illustrative quotes. We begin by summarizing results for RQ1, which address perceived acceptability of posting emotional and somatic signals, followed by summarizing results for RQ2, which summarize the signals participants reported using to identify emotional and somatic symptoms among their peers on social media.

### 4.1 Acceptability of posting emotional signals

#### 4.1.1 Self, acceptable to post.

Most interviewees said they would not personally post emotional signals on social media. Of those who did indicate it would be appropriate for them to post, it was typically boys of all ages. The responses were not a resounding yes, but rather detailed contingencies of when they would make such posts.

For some, posting was seen as acceptable when the purpose entailed sincere seeking of peer support. David (Age 14) initially replied that he would prefer not to post emotional signals on social media. When asked to elaborate on his hesitancy to post, he said, “Probably so I could probably get through it myself and try to see if I can get over it. And then if I can’t, then just post it and see who can help with it.” Scott (Age 16) described an imperative of distinguishing his post from those made by others for attention-seeking purposes, which may not be taken seriously. He explained that he needed to show “this is not for attention, this is serious,” with the hope of “find[ing] more people that are feeling this way so that way we can help each other out.” Like David, Scott in the previous quote describes seeking support, and perhaps reciprocating support between anyone feeling the same way. Jacob’s (Age 14) response mirrors the previous by both describing a need to appear serious and wanting peer support. Specifically, he said, “I make it obvious in school, and the people I have on my account if I post something like oh, I didn’t feel well today they would know already because they would see it in person, and so like maybe they would text me and be like are you okay and just have a conversation.” Thus, Jacob depicted an approach that triangulated offline and online signals.

Other contingencies included efforts to manage privacy concerns. Giselle (Age 17), the only girl who felt posting emotional signals was appropriate, specified she would post on a private story. Specifically, Giselle said, “Like if I were to be crying, I guess, I would post it...Or like if I’m sad, like, I’ll just write about it, and if like somebody slides up, I’ll just talk to them.” Just as others, Giselle noted the possibility of receiving peer support. Others noted why using private stories were important, like Jake (Age 17) who said, “Whenever I do I usually just do it with close friends…it’s usually on a private story with certain people added on to it, not with everyone…I don’t do it out in the public because I just don’t want everyone to know about me as a person like that unless I get to know them well, then that way I don’t mind them knowing about it.” Jake explains he does not want everyone to know. Similarly, the earlier quote from Jacob (age 14) expresses comfort posting on social media because he has curated his friends list to only include friends who would already know he was not feeling well based on offline interactions. The next section expands on the concerns raised here and provides a possible explanation for why interviewees tended to not endorse posting emotional signals on social media.

#### 4.1.2 Self, not acceptable to post.

The most common response to the question about whether posting emotional signals was appropriate was ‘no,’ and this was across gender and age. The girls in our sample stressed preferring alternative confidants and modalities. Jackie (Age 16) explained alternatives to posting on social media, specifically her confidants. She said, “I’ll talk to my mom or something… I usually go to certain people if I need help or if I’m feeling sad and I need help coping and just letting it out.” Lilo (Age 16) listed several others in which she would confide when she said, “I would rather have a conversation with my mom or with my therapist or with somebody like my brother that I truly confide in and not post about it on any social media.” Accordingly, not wanting to post was driven by viewing immediate family and in-person connection to confidants as superior.

Other responses echoed previously mentioned privacy concerns, except they lacked contingencies that would make it appropriate for them to post such content. These included Alfonso’s (Age 15), who said, “I wouldn’t say, like, ‘Oh, I’m depressed,’ or, like, ‘I’m sad,’ or one of those things… I wouldn’t be comfortable sharing that… it’s just personal things. I don’t want all those people knowing my business” Girls felt similarly, including Lucy (Age 14) who stated, “I wouldn’t show it because it’s something private and I don’t think there’s a need for people to see this stuff…So, even if I was sad, I wouldn’t post about it, because it’d be embarrassing. Just, it’d be embarrassing.” Both appraised posts containing emotional signals as sensitive content that should not be shared with the imagined audience of the posts, with Lucy adding she would feel embarrassed.

Reasons for reservations to personally post emotional signals included expecting to receive adverse responses. For example, after Ashley (Age 14) said, “I shouldn’t be expressing my feelings,” she explained it was “because then some people will tell you-- they’re like ‘Nobody cares’. It’s your problem. You shouldn’t be posting it. Fix it yourself.” Bob Age 17) described a similar callous response when he said, “people would use it against you or say like, ‘Stop being depressed. You’re only a kid,’” as did Archibald (Age 17) who recounted, “because I seen people who felt this way share it on social media and get made fun of.” Krystal (Age 14) echoed the others and then further clarified the types of emotions she prefers to post: “So I personally don’t show my emotions on social media unless it’s like I’m happy and like I’m going to like post a funny like picture or whatever.” Thus, while interviewees like Krystal suppressed emotional signals of depression to avoid social repercussions, they did not refrain from entirely emoting on social media.

Others, typically girls, were worried that the responses would be positive in that their peer group would be supportive, thus still refraining from posting emotional signals. For example, Anna (Age 16) said, “I don’t like to show it, because I don’t want to worry my friends about it.” Pablo (Age 17), the only boy who expressed concerns about worrying his friends, said, “I don’t want people to worry about stuff... I mean, it’s probably never serious. It’s not like suicidal kind of things,” which downplayed the significance of emotional content. Rosio (Age 16) also stated she would not post to friends and explained, “So, I wouldn’t want to show them that, ‘Oh, I’m getting sad’ just to not-- for them to get sad as well. I know some people, if somebody gets sad, then they’re sad too.” Rosio appeared to be considering that the emotions would become contagious within her peer group.

Jaden (Age 16) was likewise concerned about emotional signals being contagious, but for the original poster in the future. When asked why he would not personally post emotional signals, he replied, “think about like it in maybe a month or when, just trying and think about when you’re feeling good like would you want this out... would you want to see yourself in that position on your social media account?... you know, you can end up feeling more depressed in the future.” Because posts are archived and viewable in the future, it appears Jaden was concerned about the negative impact on his future self.

#### 4.1.3 Friend, acceptable to post.

Relative to self-posting, interviewees were more accommodating when asked whether it was appropriate for a friend to make a post with emotional signals. For example, Bri (Age 17) said, “sometimes people need a rant” suggesting she empathized with friends needing to just air their feelings. Jade (Age 13) likewise felt it was appropriate for a friend and stated, “I feel like if they just want everybody to know like, oh, like I’m not feeling like that great right now so like I don’t really want people like talking to me about it or just something like that,” suggesting such posts may serve the purpose of informing the peer group that they should halt communication.

Responses indicating it was appropriate for a friend to post emotional signals included exigencies, much as with self-posting, except these solely described the caveat of seeking support. Another difference is that the caveat did not appear to follow any gendered patterns. For example, Jackie’s (Age 17) response was, “I feel like a dramatic situation would be okay like if, let’s say, someone passed away in your family. I feel like that’s a really big issue...Because people on social media is usually your community, and so I feel like it takes a community to help with such a traumatic and dramatic situation.” David (Age 16) also described a crisis scenario, “God, I’d say like self-harm or suicidal thoughts” and indicated it was appropriate to post emotional signals “because [he felt] like that is a sign that they need help.” Less urgent scenarios were mentioned as well. Jacob (Age 15) described one, “If it’s a friend that I really care about, I would want them to share so I can talk to them” thus suggesting he himself could provide support to the friend.

#### 4.1.4 Friend, not acceptable to post.

Several reasons for feeling in was inappropriate for a friend to post emotional signals on social media parallelled those mentioned to explain why the same content was inappropriate to self-post, suggesting the power of these reasons to censor emotional signals. Specifically, these included expectations that the friend posting would receive adverse responses (the peer group not caring, expecting the person posting to fix the problem on their own, and mocking the person posting) and belief that emotional signals were private matters.

The unique reasons included concerns about the veracity of the post, particularly as Emma (Age 16) explained, “because people tend to go around saying they’re doing it for attention or they don’t feel that way, like it’s just so people feel bad for them and they get the sympathy.” David (Age 16) echoed the sentiment by saying, “Okay, the problem is nowadays teens do post stuff like that, okay like sometimes I see oh I’m going to kill myself, which is KMS, and it’s just like you just know they’re not going to do it…because on social media they say one thing but then when you see them in person they’re fine.” He questioned the veracity of such posts because they did not triangulate with what an observer sees in-person. Lucy (Age 14) also discussed self-harm in her explanation and said a friend posting it was inappropriate because “maybe other people, or maybe even younger people that you might have on Snapchat might see that and they’d be like, ‘Oh, it’s not that bad to do because they’re doing it.’” We neither observed gender nor age patterns in the reasons proffered.

### 4.2 Acceptability of Posting Somatic Signals

#### 4.2.1 Self, acceptable to post.

As with self-posts containing emotional signals, most participants hesitated to endorse posting somatic signals. However, several younger (13 and 14 year-old) girls in our sample deemed it appropriate, particularly for coping. Maui (Age 14) described seeking someone with whom to converse: “Maybe when I’m alone and I really don’t have anyone to go to, then I would, maybe, seek some type of attention.” Sara (Age 14) also explained she would post about somatic symptoms like feeling drained and being unable to sleep to reach others and explained, “then I’d need something to keep me busy to kind of forget about the feeling.” Monse Marie (Age 13) likewise said she seeks others, but also noted that just posting was relieving: “maybe if I just wouldn’t feel like talking to anybody, so just post it, I guess getting the feelings out, talking to people. “

Krystal (Age 14) also said she sought support from others. During the interview, the interviewer highlighted that Krystal seemed reticent about posting emotional signals and asked why somatic signals were different, to which Krystal responded, “A lot of people will feel the same and they’ll also want to talk about it or just text. But when you post that you’re sad not a lot of people can relate to this specific situation and you might just have to just talk to one person and they might not know what to say in that situation if they haven’t really been in it.” Accordingly, there appeared to be an expectation that, unlike emotional signals, posting somatic signals would resonate with peers because they could relate.

#### 4.2.2 Self, not acceptable to post.

As with self-posts of emotional signals, self-posts of somatic signals were typically deemed inappropriate. The reasoning mirrored emotional signals, with participants wanting to avoid worrying their peers and a sense that somatic signals were considered private, with some like Gloria (Age 17) explicitly characterizing somatic signals as “depressing stuff.” However, we did not see indications that unfavorable views of self-posts were due to preferring offline or concerns that posts would garner adverse reactions.

#### 4.2.3 Friend, acceptable to post.

As with emotional signals, interviewees appeared more accepting of somatic signals posted by friends than self-posting such content. Similar reasons were proffered, specifically to alert peers to limit communicating with the friend posting and to get support, particularly during crisis scenarios like self-harm and suicidal thoughts. Alexa (Age 14) raised a crisis scenario that was not mentioned with respect to the appropriateness of a friend posting emotional signals. She said, posting such content was appropriate if the friend were “harmed like if they’re getting hurt by anybody or anything,” indicating cases such as abuse or bullying would justify a friend posting somatic signals. Unlike when asking about the appropriateness of a self-post containing somatic signals, we did not detect patterns by gender nor age.

#### 4.2.4 Friend, not acceptable to post.

The responses expounding why it was inappropriate for a friend to post somatic signals were markedly different from all other responses. Unlike for emotional signals, crisis scenarios were not described as exigencies that interviewees deemed as warranting a friend to post. Rather, participants voiced concerns that posting was not the ideal means of obtaining help during a crisis. Anna (Age 16) explained, “if it’s a more serious situation and all that…it worries a lot of people out.” Other suggested alternatives to posing. Archibald (Age 17) stated, “if it’s a serious ‘I’m having trouble sleeping,’ like, medically, you need to go get help” and Jennifer suggested, “either like have their parents help them or like siblings.”

Like the reasoning for explaining why posting emotional signals was inappropriate for a friend, there were concerns about the veracity of the post, except the substance of the concerns differed. Maddie (Age 14) said it was inappropriate for a friend to post in the case of suicidal thoughts and self-harm because others would “feel uncomfortable” and explained, “you’re questioning them, ‘Are they okay? Do they need actual help?’” Susan (Age 16) shared similar concerns and said peers may feel confused because “you don’t really know how to help them…you text them and they say back, ‘Oh, I’m fine,’ and they’re all happy. You don’t really know what to do so you’re like, ‘Oh, okay.’ Here, the concerns about the veracity of the post suggest confusion about whether someone should intervene and offer support.

### 4.3 Signals used by latino adolescents to classify depressive symptoms

Table 2 summarizes responses to questions asking interviewees how they can tell from a friend’s social media account when the friend may be experiencing symptoms consistent with depression. We organized responses by symptom (emotional, somatic) and the type of data (metadata, literal statements, changes in text, audiovisual assets).

**Table 2:** Signals for emotional and somatic symptoms.

Type of data	Signal	Examples by Set of Symptoms	
		Emotional	Somatic
Metadata	Time taken to reply	(not applicable)	Martin John (Age 15): I had friends just not reply for, like, five hours.
	Changes in frequency of posting	John (Age 16): Maybe if it’s somebody that posts a lot then if they start posting less. Or sometimes even more than. It’s really weird because there’s different types of people out there.	Jacob R (Age 15): So if it’s somebody that posts a lot you can tell that they’re not feeling it if they’re not posting as much.
	Time stamp	(not applicable)	Gloria (Age 17): Maybe like the time that they post it, too, because that really tells a lot, because like they might be up at 3:00 in the morning…like you know they can’t sleep.
	Likes, following, reposting	Lilo (Age 15): Like start to like like a whole bunch of quotes like that, or like sad accounts, or like stuff like that...there’s just like some accounts on Instagram that have-- they’re like-- it’s called like sad quotes, and they’re just like a whole bunch of quotes about like feeling really down and feeling really helpless and hopeless.	(not applicable)
Literal statements	Depression	Alexa (Age 14): They usually just say, “I’m depressed”	(not applicable)
	Symptom of depression	Bri (Age 17): What they post. I know someone who was, “I’m a failure. I can’t. I can’t do this no more.” They were saying about they were struggling in school.	Emma (Age 16): When they post “can’t sleep” or “having trouble sleeping,” something like that, it’s letting people know they’re up
	Context	Monse Marie (Age 13): yeah, when they missed a parent that was in jail at the time, prison...they were just very emotional at the time...posting pictures about the person, saying how much they missed them, things like that.	Jamal (Age 15): they mostly, I guess, complain…they say, like, “I have this much to do and this much to do, and it just keeps going and going and going,” and I think that’s a little easier for me to recognize like, “Oh, they’re really feeling this way.”
Changes in text	Linguistic shift	David (Age 14): Like they start commenting or texting the way they don’t usually…like some people actually respond in lengthwise, and then sometimes when they’re sad they just do little responses.	Jacob (Age 14): Like if it’s someone that I text on the daily and they just start texting dry and like texting differently… like just short words…that’s when I would ask if they’re okay or if something’s wrong.
	Mood shift	Lisa (Age 16): Like if that friend normally posts stuff about good, positive things, or like-- and they suddenly just post this sad-- something sad. I’d be like, yes, yeah, that something’s up.	Dahlila (Age 14): If they’re usually posting like happy things or funny things, then they’ll like stop posting those things and they’ll post I don’t know other stuff.
	Profile description	Alfonso (Age 15): Well, the same friend said that under her profile she said she was dead inside, so I was shocked, I guess. And then she usually change it to, like, how she’s feeling. Sometimes she’d put, “I’m feeling blue,” or, “I feel sad,” or something like that.	(not applicable)
Audiovisual assets	Quotes	Lilo (Age 15): Yeah. I feel like sometimes people like post like some quotes. They post some like really sad quotes about like you know, like life or how they’re feeling, without, you know, saying, like, oh, I’m feeling this way.	(not applicable)
	Emojis	Alexa (Age 14): They usually like add emojis…Heartbreak emojis. And they usually say it, too. They’re like, “Heartbroken.”	Ashley (Age 14): Like depending on the emojis they use, like they can use the like the crying emoji or the mad emoji, they use the skull...there’s like this one on iPhone...but it’s like where you’re like sad and you’re blowing your nose like if you’re sick.
	Selfies	Jaden (Age 16): I try and, I don’t know, like kind of like read their body language I guess, like in their emotion in the picture, like how’s their face like? Are they smiling? Are they-- do they look positive? If not, do they look like angry? Do they look sad? You know, you kind of read what they look like.	Susan (Age 16): Maybe just that same girl keeps posting a picture of her crying or just a cold saying like, “I give up.” Or her physically taking a picture of her and crying, and actually writing I give up.
	Stickers	Jackie (Age 17): They’re usually these little emoji stickers…a sad face, a crying face. Sometimes the stickers move around and be like-- some of them have phrases on them.	Emma (Age 16): Maybe by posting a little sticker or something that represents sleep, and maybe the time it is.
	Videos of themselves	Ashley (Age 14): I haven’t dealt with any of my friends being like that, but they would send me videos of them crying if they’re sad or them-- my God-- of them like telling me -- like a video of them being mad, like they’re frustrated, you can tell.	Maria (Age 14): So they put themselves in the video like crying-crying.
	Songs	Dahlila (Age 14): Sometimes people will post songs or like they’ll caption things with like I’m feeling sad or something like that.	(not applicable)
	Bitmoji	John (Age 13): Maybe change their name, I guess. Or there’s this little feature. It’s a bitmoji where you change-- it’s like a little you. They maybe can change their look or just take it off to give their account a more depressing look.	(not applicable)
	Color	Lucy (Age 14): It usually looks dark, like goth kind of, and that’s bringing-- that all connects to the whole goth aesthetic or just, I don’t know.	(not applicable)

Metadata refers to information about a post, such as the time that the post was made. Literal statements include both explicit statements stating they are depressed or experiencing symptoms consistent with depression, as well as statements suggested experiencing such symptoms, such as describing a stressful context. Changes in text refer to observed changes in the text from the friend, such as a change in the typical mood they convey. Audiovisual assets include songs, videos, photos, and emojis.

Several differences are seen between the two symptoms. More types of metadata were mentioned for somatic versus emotional symptoms. The only type of metadata that was mentioned for emotional symptoms but not for somatic was likes, following, and reposting. Conversely, more types of audiovisual assets were mentioned for emotional versus somatic symptoms. Quotes, bitmojis, songs, and the color of the post were mentioned for emotional symptoms, but not for somatic symptoms. Such audiovisual assets seemed integral to apprehending emotion.

Where the two symptoms appear most similar is in the types of literal statements made by the friend. Each type of literal statement appeared for both symptoms, with the only exception being the literal statement of depression, which was only mentioned for emotional symptoms. Another similarity is in the use of selfies and videos as audiovisual assets, which seemed to be used to understand the experience of the symptoms, such as crying.

## 5 Discussion

To address the lack of inclusion of adolescent perspectives in the development of ML models to detect depression within social media data, we took a critical step by adopting an adolescent-centered approach. Specifically, we conducted interviews with Latino adolescents, who prior research suggests exhibit high levels of somatic symptoms, reflective of the patterns of depressive symptoms among adolescents more broadly. We sought to identify their norms regarding sharing content indicative of emotional and somatic symptoms of depression (RQ1) and what such content possibly looks like (RQ2). We found evidence suggesting norms differed between the two sets of symptoms, with norms appearing more strict for sharing emotional symptoms and norms seeming to vary across gender and age. Prescriptions against posting emotional symptoms seemed to relax when the person posting needed help, particularly for a crisis. The data used by interviewees to detect both sets of symptoms evinced differences, but also some similarities, a notable one being the variety of signals used beyond just literal statements of depression (e.g., “I’m depressed”). We discuss the implications of these findings for future steps in developing ML methods to detect depression among adolescents and the utility of centering on the experiences of Latino adolescents.

We found evidence suggesting that the norms about sharing posts conveying emotional and somatic symptoms differed. Posting emotional signals appears particularly restricted because most interviewees said they would not themselves post, but it tended to be boys who said they would post because they felt they could access support. Among girls, they seemed to prefer approaching their confidants in-person. While it is difficult to form demographic generalizations from the small sample, the patterns accord with extant research. The gender difference may explain why, despite masculine ideals prescribing against emotional expression, other work suggests boys are more likely than girls to make distressing posts [38]. Masculine ideals may still be operant, in that hegemonic forms of masculinity typically restrict men from having confidants [42], which may explain why boys depend on social media more so than girls for support. Across genders, interviewees appeared more accommodating of a friend posting, suggesting restrictions against posting could ease to help a peer. However, there were concerns expressed when describing self- and friend-posting about adverse responses. When describing friends posting, interviewees appeared concerned that others would question the validity of the post. It appeared important to communicate the context around the emotions, perhaps because emotions are only considered deviant if they conflict with the context [43,44].

Regarding the norms about posting somatic signals, we observed differences when we asked about self- versus friend-posting. Somatic self-posting appeared appropriate only among the younger (13 and 14 year-old) girls in our sample, suggesting norms differ by age. This aligns with research indicating that among adolescents, girls are more likely than boys to express somatization and somatization is more common among younger compared to older adolescents [31,34,35]. A rationale appears to be that somatic symptoms are more relatable to others than emotional symptoms, but this may disappear as adolescents grow older. Unlike with emotional symptoms, we did not see any concerns about hostile responses to posts conveying somatic symptoms. This may be because somatic symptoms appeared to be associated with crises, including harm (both self-harm and from others). The findings suggest the need to develop and validate ML methods to use the identified signals, particularly those indicative of somatic symptoms, to detect depressive symptoms within adolescents/ social media data.

Toward that end, we identified a heterogenous set of potential signals for both emotional and somatic symptoms (RQ2), which future research can further investigate to validate their clinical significance and suitability for feature engineering. Literal statements, while common in some ML methods, were not the only signals mentioned. Perhaps because of the unfavorable views of making posts with emotional signals, adolescents avoid making literal statements and instead use other affordances, like audiovisuals to communication emotions. Other signals for emotional symptoms included videos and selfies, which may be to enhance credence to their posts and avoid being labeled as an “attention-seeker” [28,33]. Thus, the norms against posting content conveying emotional symptoms may not preclude conveying the information, but rather encourage creativity in conveying the information, thereby still making it possible to detect emotional symptoms. Metadata appear promising, particularly for detecting somatic symptoms. Prior work has investigated incorporating some of the metadata we identified in the current study, such as posting behavior [22] (e.g., posting during twilight hours or posting less), but did not find it to significantly predict the onset of depression. Changes in posting behavior, such as posting less, may also indicate better mental health [45], and thus the signal may not perfectly correlate with symptoms. Additionally, because the population analyzed was adults, the findings from the current study suggest the need to revisit posting behavior within the adolescent population as a signal. Altogether, the documentation of multi-modal expressions of depressive symptoms (including audiovisuals and metadata) is critical to consider in feature engineering to develop ML methods targeted toward adolescents, because the current study suggests analyzing only text (particularly for literal statements) appears inadequate.

Results should be understood in the context of the study’s limitations. The responses may be subject to recall and social desirability bias, because we did not ask interviewees to share their social media feed with us. We did not do so to preserve the privacy and confidentiality of those in their social media networks, particularly because we were made aware that some interviewees and their families were undocumented immigrants to the U.S. The design was developed to present scenarios and determine norms, but there may be instances in which adolescents may opt to deviate from them. Future research should pursue safeguards to observe the social media feeds of adolescents and elaborate on our findings. Another limitation is that the sample is from one Midwestern city and is comprised of mostly Latinos of Mexican origin who preferred to be interviewed in English, which limits representation of the Latino adolescent population and generalizability. More research is needed to identify potential subgroup differences within Latino adolescents and language differences. The lack of men among the interviewers may have introduced some biases, such as who enrolled or what was shared. Lastly, we did not randomize the order in which we asked about emotional and somatic signals, which may have led to priming effects. This would likely have the effect of overrepresenting similarities between the two signals.

Despite these limitations, this study is a critical step toward designing and developing mental health informatics interventions for adolescents. By centering on Latino adolescents and asking their lived experiences on social media with both emotional and somatic symptoms of depression, we identified opportunities to fine tune the automated detection of depression within social media data. We echo the calls of others for more research documenting differences in depression profiles and norms related to online disclosures and help-seeking among adolescents [46], which would aid in enhancing the detection of depression within user generated content. The next step is building from the signals we identified through our study for feature engineering, such as including signals for somatic symptoms like audiovisual cues and posting behavior and validating them. This entails determining the specific types of songs, quotes, and images that are likely associated with depressive symptoms within an adolescent user population and then comparing how well these correlate with symptoms compared to extant methods, like using users’ literal statements or a medical diagnosis. Because specific cues are likely to shift over time, it will be critical to develop frameworks for revising features, such as our adolescent-centered approach. A consideration of age and gender in a large sample of adolescents would also be warranted. While initial evidence suggests that adolescents are receptive to social media platforms seeking outside help based on symptoms shared online, additional work is needed to identify the acceptability of applying such ML methods to adolescents, particularly given the privacy concerns raised regarding the content. Such steps are critical to address the growth of mental health concerns among adolescents.
